# The cascading pathogenic consequences of *Sarcoptes scabiei* infection that manifest in host disease

**DOI:** 10.1098/rsos.180018

**Published:** 2018-04-18

**Authors:** Alynn M. Martin, Tamieka A. Fraser, John A. Lesku, Kellie Simpson, Georgia L. Roberts, Jillian Garvey, Adam Polkinghorne, Christopher P. Burridge, Scott Carver

**Affiliations:** 1 School of Natural Sciences, University of Tasmania, Sandy Bay, Tasmania 7005 Australia; 2 Animal Research Centre, University of the Sunshine Coast, Sippy Downs, Queensland 4556 Australia; 3 School of Life Sciences, La Trobe University, Melbourne, Victoria 3086 Australia; 4 School of Humanities and Social Sciences, La Trobe University, Melbourne, Victoria 3086 Australia; 5 Department of Primary Industries, Parks, Water and Environment, Hobart, Tasmania 7000 Australia; 6 School of Science and Engineering, Federation University, Mount Helen, Victoria 3350 Australia

**Keywords:** *Vombatus ursinus*, sarcoptic mange, pathophysiology, metabolic rate, fatty acid composition, time budget

## Abstract

Sarcoptic mange, caused by the parasitic mite *Sarcoptes scabiei*, causes a substantive burden of disease to humans, domestic animals and wildlife, globally. There are many effects of *S. scabiei* infection, culminating in the disease which hosts suffer. However, major knowledge gaps remain on the pathogenic impacts of this infection. Here, we focus on the bare-nosed wombat host (*Vombatus ursinus*) to investigate the effects of mange on: (i) host heat loss and thermoregulation, (ii) field metabolic rates, (iii) foraging and resting behaviour across full circadian cycles, and (iv) fatty acid composition in host adipose, bone marrow, brain and muscle tissues. Our findings indicate that mange-infected *V. ursinus* lose more heat to the environment from alopecia-affected body regions than healthy individuals. Additionally, mange-infected individuals have higher metabolic rates in the wild. However, these metabolic demands are difficult to meet, because infected individuals spend less time foraging and more time inactive relative to their healthy counterparts, despite being outside of the burrow for longer. Lastly, mange infection results in altered fatty acid composition in adipose tissue, with increased amounts of omega-6 acids, and decreased amounts of omega-3 acids, a consequence of chronic cutaneous inflammation and inhibition of anti-inflammatory responses. These findings highlight the interactions of mange-induced physiological and behavioural changes, and have implications for the treatment and rehabilitation of infected individuals.

## 1. Background

The condition of ‘disease’ conferred upon hosts by infectious organisms is a manifestation of cascading pathogenic effects following infection, the summation of which can translate to effects on population, community and ecosystem scales. Yet, for many important wildlife diseases, the cascades underscoring disease manifestations remain poorly understood. This is particularly consequential where the host range of infectious organisms may be expanding, such as is often the case with emerging infectious diseases. Importantly, understanding the cascading consequences of infection can provide insights across existing and new host species for the development of strategies for the treatment and rehabilitation of individuals, as well as the prevention and management of disease transmission.

*Sarcoptes scabiei* is a globally widespread parasitic mite and the aetiological agent of sarcoptic mange disease in humans (scabies), domestic and wild animals [1,2]. Mange is among the 30 most common human infectious diseases with an estimated 300 million cases annually, and was recently listed by the World Health Organisation as a ‘Neglected Tropical Disease’ [3,4]. This parasite causes a significant burden of disease, and has been documented to infect more than 104 mammal species, spanning seven families [1,2]. Mange is known to be particularly severe to some host species, including *Vulpes vulpes* (red fox), *Capra pyrenaica* (Spanish ibex) and *Vombatus ursinus* (bare-nosed wombat) [5–7]. The global host range of *S. scabiei* continues to expand, and represents a significant emerging infectious disease [8].

**Figure 1.**
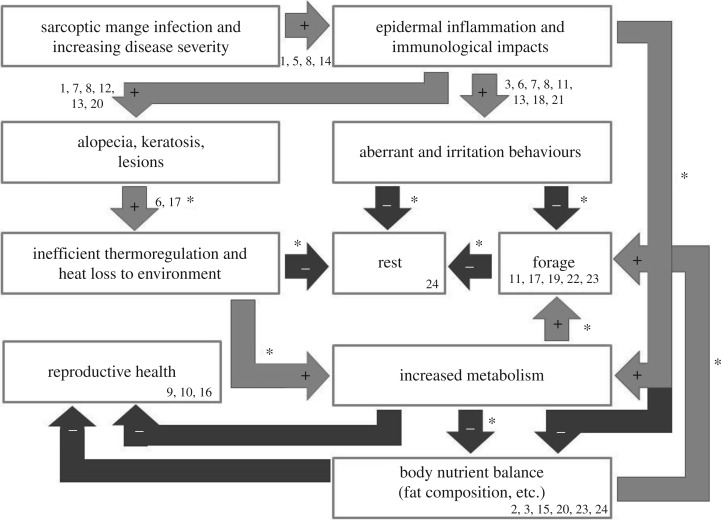
Cascading impacts of *S. scabiei* infection causing mange disease manifestation. (*) Denotes disease associations investigated in this study. (1) Arlian & Morgan [9], (2) Beigh *et al.* [25], (3) Beigh *et al.* [26], (4) Borchard *et al.* [24], (5) Bornstein *et al.* [2], (6) Cross *et al.* [18], (7) Cypher *et al.* [27], (8) Diwakar & Diwakar [28], (9) Fthenakis *et al.* [10], (10) Laha [11], (11) Murray & St Clair [29], (12) Nimmervoll *et al.* [30], (13) Oraon *et al.* [31], (14) Pence & Ueckermann [1], (15) Pérez *et al.* [32], (16) Sarasa *et al.* [12], (17) Simpson *et al.* [19], (18) Skerratt *et al.* [33], (19) Skerratt *et al.* [34], (20) Skerratt [35], (21) Süld *et al.* [36], (22) Süld *et al.* [37], (23) Tataruch *et al.* [38] and (24) Verstegen *et al.* [39].

Infection by the *S. scabiei* mite confers a diverse array of impacts on hosts. Clinical signs of mange are often observed four (or more) weeks post exposure, when the mites have established a population on the host [9]. During the early stages of infestation (the first few weeks), mites are able to suppress the host immune response. However, as mite densities increase, the host begins to exhibit an initial hypersensitivity reaction resulting in skin inflammation [1,2,9], causing a suite of effects that fall into two broad categories: physiological and behavioural. Physiological effects encompass changes in metabolism, skin condition [1], reproduction [10–12], biochemistry [13–15], growth [16,17], thermoregulation [18,19], immunology [20] and body condition [17,21]. Behavioural effects include irritation [1], as well as changes in foraging [19], home range and dispersal [7,22], and circadian rhythmicity [23,24]. These effects have complex interactions, whereby one disease condition can cause, influence or amplify another, resulting in cascading and compounding pathogenic impacts on the host (figure 1).

Although a range of characteristics connecting *S. scabiei* infection to mange disease is known, important knowledge gaps remain. Evidence suggests that the energetic burden of mange must be substantial, as weight loss is commonly observed in mange-infected hosts [1,2]; however, the metabolic impacts of *S. scabiei* infection are poorly understood. It is possible that *S. scabiei* infection may increase host metabolism owing to the costs of mounting an immune response. Evidence also suggests that there may be metabolic costs of alopecia and associated thermoregulation [18,19], but investigations regarding this topic remain limited. The host may attempt to compensate for the aforementioned changes through shifts in behaviour (e.g. to save energy or increase energy intake). However, evidence is inconsistent about the nature of these shifts [19,39], and information is needed that encompasses full circadian rhythms. *Sarcoptes scabiei* infection can result in a loss of animal condition through depletion of fat stores [17,21,40], but infection may also cause an imbalance of fatty acid composition across tissues (e.g. brain, bone marrow and muscle tissues), that contributes to function (e.g. immune function, motor skills, behaviour), which is less well recognized.

Wombats are an important example of a host species for which *S. scabiei* has become an emerging disease, and continues to cause significant pathology [5,41]. Consistent with other host species experiencing crusted mange disease, infected wombats experience skin fissures and hyperkeratosis [35,42,43], reduced fat stores and emaciation [21,44,45], loss of body condition [21,33,46], decreased reproductive function [21,46] and higher thermal differentials [19]. Behaviourally, they exhibit increased diurnal activity [21,24,43], may travel farther [33], spend more time outside of the burrow [19], and reallocate the amount of time devoted to different behaviours [19].

Here, we develop the body of knowledge around the cascading consequences of mange disease manifestation, focusing on the Tasmanian bare-nosed wombats (*V. ursinus*). We address critical knowledge gaps linking the cascading effects of infection that manifest in disease, broadly encompassed within the disciplines of integrative biology and conservation physiology. Specifically, we aim to: (i) quantify heat loss in mange-infected wombats, (ii) calculate field metabolic rates for diseased and healthy individuals, (iii) assess behavioural changes across full circadian cycles, specifically foraging and resting behaviours, and whether these are sufficient in compensating energy demands of disease and, (iv) quantify fatty acid composition across tissues in mangy wombats. Owing to the challenges associated with obtaining these types of physiological and behavioural data in free-living animals, we have exploited multiple data sources. Our sample sizes are necessarily moderate, but provide valuable insight to the powerful impacts of *S. scabiei* on its hosts.

## 2. Methods

### 2.1. Scoring of mange severity

Mange severity scoring followed the protocol described by Simpson *et al.* [19] whereby each individual is divided into 14 body segments, seven segments on each side: head (H), shoulder (Sh), forelimb (FL), stomach (St), back (B), hind limb (HL) and rear (R). Each segment is assigned a score reflecting hair loss, from 0 (no hair loss) to 10 (greater than 70%) (electronic supplementary material, A). The average mange severity score is the mean of the segment scores (with the exception of §2.2, where mange severity is the average of the FL, St and HL only). Owing to the asymmetric nature of alopecia in mange-infected wombats, averaged mange severity scores are typically much lower than the most severely infected individual segment score. Mange severity scores ranged from completely healthy (lowest severity score: 0) to late-stage mange (highest severity score: 7.5) [41].

### 2.2. Aim I: quantifying heat loss

To understand the energetic cost of mange-induced hair loss on wombat thermoregulation, we used thermal imagery to calculate heat loss (W m^−2^). Thermal imaging has been a powerful and non-invasive tool in studying thermoregulation and thermal physiology, performing population surveys and count surveys, and diagnosing disease [47]. This method has been used to both diagnose [48] and quantify the impact [18] of sarcoptic mange in wildlife.

**Figure 2.**
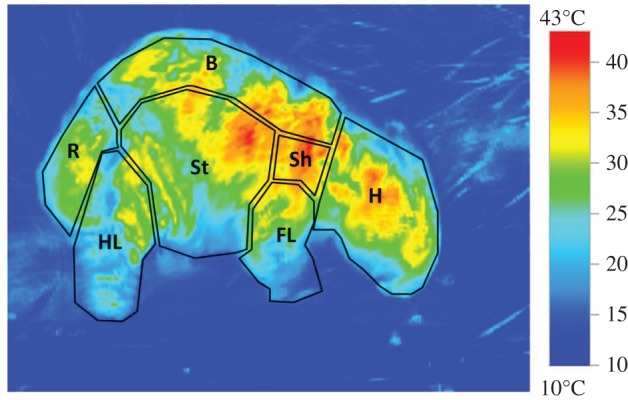
Thermal image of a wombat with the seven segments defined. Each segment was assigned a mange score based on methods described in Simpson *et al*. [19], and the maximum temperature from each segment was documented. Forelimb (FL), stomach (St) and hind limb (HL) were used for heat loss analyses, while rear (R), back (B), shoulder (Sh) and head (H) were excluded.

Five free-living wombats at Narawntapu National Park (NNP; Tasmania, Australia, 0466482 E, 5444789 N) were opportunistically photographed using the Testo 870-1 thermal imager (thermal sensitivity less than 0.1 K, 32° field of view, 320 × 240 pixels) from March to June 2014 (electronic supplementary material, B). Individuals ranged from healthy (highest individual segment scores of 0–2, *n* = 2) to late-stage mange-infected (highest segment scores of 9–10). Photographs were taken manually, with replicate photographs taken of each individual to capture various angles (with profile angles being optimal for viewing all body segments; figure 2). Hourly ambient temperature was collected by the nearest weather station in Devonport, Tasmania (Devonport Airport, 0451985 E, 5442007 N; 10 km east of NNP), and ranged between 9.7°C and 18.9°C. Images were processed using software (Testo IRSoft v. 3.1) to calculate maximum surface temperatures for each body segment. The temporal span of photos of healthy and mange-infected wombats overlapped, such that any effects of mange are not confounded by time of day. Indeed, most images were taken during the evening. Owing to the diurnal behaviour of mange-infected wombats [19], some images were necessarily taken during daylight hours; many others in overcast weather. Also, to avoid possible impacts by solar radiation, the Sh, B and R segments were not used for analyses. Regardless, hair loss in mange-infected wombats is predominately observed in the FL, St and HL segments, and thus the omission of the Sh, B and R from analyses was inconsequential for examining the thermal energetic costs of *S. scabiei* infection. Average mange severity scores for this analysis were the average of the mange scores from the FL, St and HL (figure 2). Heat loss was defined as the sum of convective (free and forced) and radiative heat loss, following methods by Cross *et al.* [18] (electronic supplementary material, C). Linear regressions were used to understand the relationship between mange score (balding %) and heat loss (W m^−2^) for the FL, St and HL segments. Heat loss (W) can be transformed into kJ h^−1^ per segment using the segment area and the conversion of 1 W to 3.6 kJ h^−1^. Total heat loss (per hour) for each wombat is the sum of heat loss in the FL, St and HL.

### 2.3. Aim II: field metabolic rates

The doubly labelled water (DLW) technique was used to estimate field metabolic rates and water turnover rates in wombats [49–51]. Our methodology followed the two-sample DLW protocol whereby: (i) a background blood sample is taken; (ii) the body water pool is enriched with hydrogen and oxygen isotopes (equilibration); (iii) a second blood sample is taken after isotope equilibration with body water pool; and (iv) a final blood sample is taken after one to two biological half-lives of the oxygen isotope. Carbon dioxide production can be estimated from the amount of isotope depleted over time [52].

A total of nine individuals (eight adults, one juvenile) were trapped at NNP between April and June 2015 to assess field metabolic rates (table 1). Individuals were trapped on foot using large, mesh nets and were anaesthetized (zolazepam/tiletamine, Zoletil, Virbac, dose: 3–4 mg kg^−1^ and medetomidine, dose: 40 µg kg^−1^ intramuscular (IM) injection; ethics approval permits A14670, FA15122). Individuals were weighed and the initial blood draw was taken, followed by an intra-peritoneal injection (IP) with 4 ml of ^18^O (greater than or equal to 98%) and 4 ml of deuterium (greater than 99.9%) following Evans *et al.* [51]. Wombats remained under light anaesthesia (with re-administration of zolazepam/tiletamine and medetomidine on a per wombat basis) during the equilibration period after the initial blood draw and isotope injection, and through the second blood draw (taken approx. 4 h after isotope injection and approx. 4.5 h post initial blood draw) [51]. Post-processing, wombats were administered a sedative reversal (atipamezole, dose: 40 µg kg^−1^ IM) and held in wire Mascot animal traps, padded and insulated with hessian sacks, for 6–12 h until fully recovered from anaesthesia. Wombats were released at the site of capture. Recapture efforts for final blood draw were focused one to two ^18^O biological half-lives after initial capture (10–14 days [51]). Eight of the nine individuals were recaptured for the final blood draw, all eight to 13 days after initial capture.

**Table 1.** Water turnover, metabolic and feeding rates of *Vombatus ursinus* (*n* = 8). (Averages are calculated for adults grouped as healthy (highest segment score ≤ 2) and early disease stage (highest segment score ≥ 3).)

animal					BM	total body water		water turnover		field metabolic rate			feeding rate
ID	sex	RS^a^	age	average mange severity score	*g*	*g*	%	efflux ml kg^−1^ d^−1^	influx ml kg^−1^ d^−1^	ml CO_2_ g^−1^ h^−1^	kJ d^−1^	kJ kg^−1^ d^−1^	g plant matter d^−1^
10	F	n.a.	J	0.00	8000	5536.2	0.69	92.04	98.39	0.363	1609.12	189.31	217.45
11	F	R	A	0.14	19 500	13100.1	0.67	86.90	86.90	0.288	2925.43	150.02	395.33
12	M	n.a.	A	0.00	26 000	19253.7	0.74	119.92	118.59	0.286	3833.08	148.86	517.98
17	F	n.a.	A	0.64	21 000	16556.3	0.79	130.87	134.99	0.319	3575.38	166.30	483.16
18	F	R	A	0.14	19 000	13269.9	0.70	59.40	59.40	0.272	2690.53	141.61	363.59
22	F	n.a.	A	1.00	22 000	15736.5	0.72	57.71	55.61	0.327	3700.07	170.12	500.01
mean								90.96	91.10	0.298	3344.90	155.38	452.01
s.e.								15.08	15.75	0.01	226.02	5.47	30.54
16	M	n.a.	A	1.61	20 000	15195.6	0.76	123.12	123.12	0.485	5052.64	252.63	682.79
19	F	R	A	1.68	21 000	15787.9	0.75	49.07	49.07	0.353	3862.21	183.91	521.92
mean								86.09	86.09	0.419	4457.42	218.27	602.35
s.e.								37.03	37.03	0.07	595.21	34.36	80.43

^a^Reproductive status (R represents a reproductive adult).

Blood samples from the initial (background and post-equilibration) and final captures were sent for deuterium and ^18^O enrichment analyses (Metabolic Solutions, Inc, New Hampshire, USA). Enrichments were used to calculate water flux and CO_2_ production (ml CO_2_ g^−1^ h^−1^) using equations from Nagy [52]. Field metabolic rates (kJ d^−1^) were calculated from CO_2_ production using the factor 21.8 J ml^−1^ CO_2_, which was derived from a koala leaf diet [51,53]. Lastly, feeding rates (required amount of dry plant matter intake to meet metabolic needs) were calculated using the metabolizable energy available through the wombat diet, which is estimated to be 7.4 kJ g^−1^ [51]. A linear regression was used to understand the relationship between mange severity score and metabolic rate. The one juvenile wombat captured was not included in the linear regression analysis, owing to different energetic requirements for juveniles relative to adults.

### 2.4. Aim III: resting and foraging behaviour

To assess disease-induced behavioural differences in wombats, triaxial accelerometer data loggers (AX3 Axivity) were deployed on five adult, free-living wombats in NNP (one healthy, one with ambiguous signs of early mange and three mange-infected). Wombat trapping and processing followed the protocols outlined above (see §2.3). All loggers were set out within 24 h of each other, on 20–21 April 2015. Three loggers were successfully retrieved: one from a moderately mange-infected wombat (female,W006, mange severity score 2.7), one from a wombat with ambiguous signs of early mange (male, W009, mange severity score 0.57, referred to as ‘early’), and one from a healthy wombat (female, W002, mange severity score 0.5). Despite having a similar average mange score to W002, W009 had ambiguous signs of early mange at capture (with confirmed mange in subsequent visual surveys), and thus, was conservatively classified as early-stage mange. The loggers recorded at 50 Hz from noon on 22 April 2015 to varying times on 18 May 2015.

Traces of the three cardinal axes of the accelerometer were visualized in Somnologica Studio 3.0 and activities were defined based on stereotypic patterns. To calibrate real-time wombat activities with accelerometer recordings, Axivity data loggers were also deployed on two healthy, captive wombats. Based on captive wombat accelerometer recordings, six main activities were identified: digging, steady walking, scratching, running, slow walking/grazing and inactivity. In addition, there were four unidentified activities and an activity categorized as ‘restlessness’, which was defined as a period of brief, unrecognizable activity interrupting periods of inactivity. Activities were manually scored in 3 s epochs (28 800 epochs per day), and each epoch was categorized as the activity that endured for the majority of that epoch. Activities were scored for four, 24 h periods (at 3 day intervals, excluding the first 72 h post-anaesthesia: 24 April, 27 April, 30 April, 3 May) for each wombat (115 200 epochs individually scored per wombat or 345 600 total).

Inactivity and foraging behavioural data were quantified in three ways: total number of episodes per behaviour (per day), average duration of activity bouts and percentage of day spent engaged in either state (for daily activities and averages, see the electronic supplementary material, D and E). For the average duration of activity bouts, bouts were defined as either an isolated epoch of activity (3 s) or consecutive epochs of the same activity (greater than 3 s). Differences in the number of episodes, bout durations and the proportion of time spent engaged in inactivity and foraging were analysed among wombats using ANOVAs. Inter-individual differences were assessed using a multi-comparison of means (Tukey contrasts). Plots of daily wombat activity from 12.00 on 22 April to 05.00 on 8 May show differences in circadian cycles (electronic supplementary material, F).

To assess whether mange-infected wombats can cope with the metabolic pressures of mange, realized feeding rates (energy consumed (kJ d^−1^), derived from behavioural data) were also calculated. The average proportion of the day spent foraging (for the healthy and late-stage wombats) was used, in combination with bite rates derived from Simpson *et al*. [19] for healthy wombats and mange-infected wombats, to determine the number of bites taken per day. The amount of dry plant matter per wombat bite was assumed to be 0.015 g, along with 7.4 kJ metabolizable energy per gram of dry plant matter [51].

### 2.5. Aim IV: fat composition

Fatty acid composition was analysed in four tissue types (adipose, brain, bone marrow, muscle) from eight Tasmanian wombats, which were euthanized owing to injuries from vehicle collision or severe mange disease, between 2015 and 2016 (electronic supplementary material, B). Wombats were given a mange score and condition assessment prior to sampling. Average mange severity scores ranged from 0 (completely healthy) to 7.5 (late-stage mange infection). Muscle was obtained from the shoulder region, bone marrow from the femur and adipose subcutaneously.

Fat composition profiles were calculated by the National Measurement Institute (NMI) for each tissue type using fatty acid methyl esters (FAME). Proportions of FAMEs are relative to the amount of sample tissue (0.5–10 g) and were determined by gas chromatography. Principle component analyses (PCA) were performed for each tissue type to select for fatty acids that had the best explanatory power (loading values greater than or equal to 0.3). Linear regressions were then run for each of the four tissues, using the results from PC1 and the mange severity scores.

## 3. Results

### 3.1. Aim I: sarcoptic mange and heat loss

**Figure 3.**
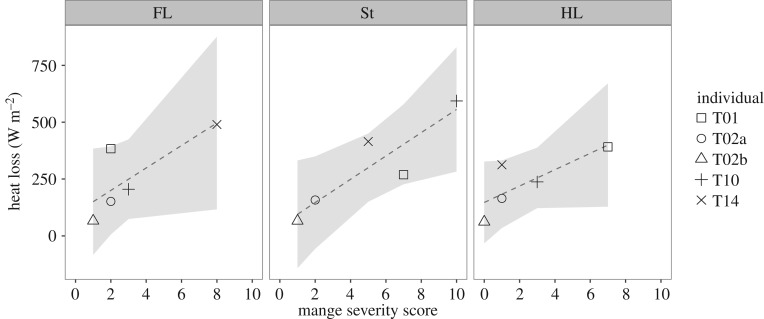
Heat loss (W m^−2^) from three body segments in healthy and mange-infected wombats (*n* = 5) with 95% CIs (grey). As the segment mange score increased, the amount of heat lost increased in the FL (*R*^2^ = 0.50, *F*_1,3_ = 5.01, *p* = 0.11), St (*R*^2^ = 0.75, *F*_1,3_ = 12.74, *p* = 0.04) and HL (*R*^2^ = 0.49, *F*_1,3_ = 4.91, *p* = 0.11). Individuals are identified to show the variation in mange severity across body locations, reflecting infection asymmetry and inter-individual variation.

Mange severity scores varied within individual wombats across body segments (figure 3). All body segments showed a positive relationship between mange severity and heat loss, with stronger relationships shown in the St (*R*^2^ = 0.75, *F*_1,3_ = 12.74, *p* = 0.04), and more moderate relationships for the FL (*R*^2^ = 0.50, *F*_1,3_ = 5.01, *p* = 0.11) and the HL (*R*^2^ = 0.49, *F*_1,3_ = 4.91, *p* = 0.11; figure 3).

### 3.2. Aim II: mange and host water flux and field metabolic rate

**Figure 4.**
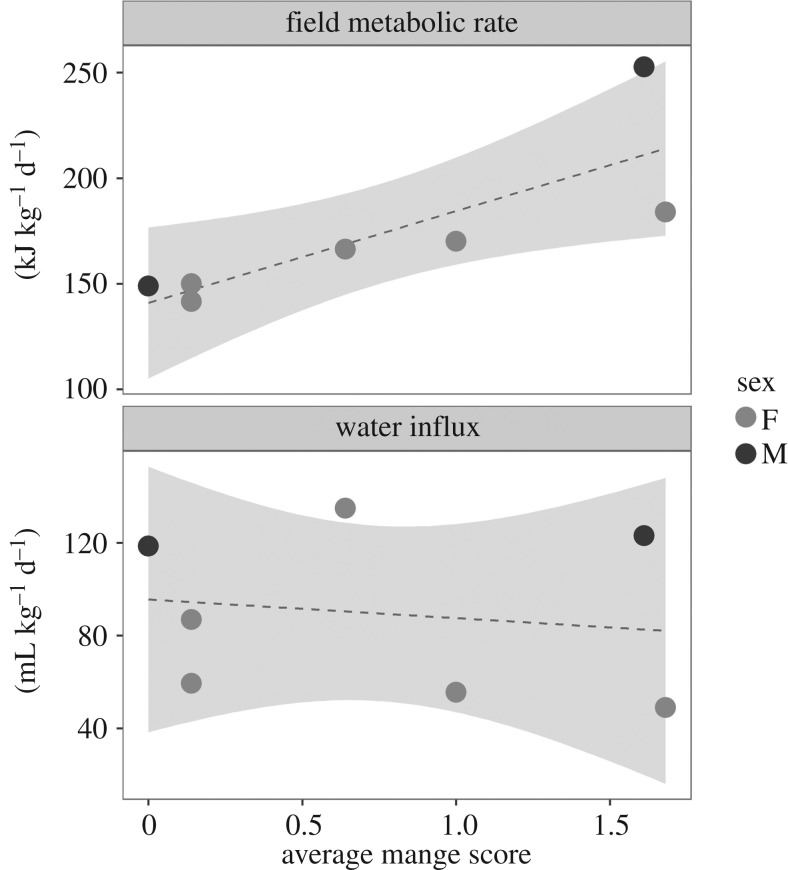
The effect of mange severity on wombat field metabolic rate (kJ kg^−1^ d^−1^). Seven adult wombats (five female, two male) at varying mange severities were used to assess field metabolic rate. The average mange severity score is the mean of the body segment scores, as described by Simpson *et al*. [19]. As average mange severity increased, metabolic rate increased (*R*^2^ = 0.59, *F*_1,5_ = 9.57, *p* = 0.03). This trend holds true when females are analysed separately (*R*^2^ = 0.89, *F*_1,3_ = 36.38, *p* < 0.01).

There was a significant positive relationship between average mange score and metabolic rate (kJ kg^−1^ d^−1^) (*R*^2^ = 0.59, *F*_1,5_ = 9.5, *p* = 0.03; figure 4). This trend continues to hold true when the regression is run with adult females only (*R*^2^ = 0.89, *F*_1,3_ = 36.38, *p* < 0.01). No significant relationship was observed between average mange score and water influx (ml kg^−1^ d^−1^) (*R*^2^ = –0.17, *F*_1,5_ = 0.13, *p* = 0.74). On average, individuals infected with mange experienced a 40% increase in their field metabolic rate compared with healthy wombats (155.4 kJ kg^−1^ d^−1^, 218.3 kJ kg^−1^ d^−1^, respectively).

Based on the results and assumptions of food calorific content, healthy wombats (with a highest segment score of less than or equal to 2; *n* = 5) would need to consume 452 g of plant matter (±30.54 g) per day to meet their metabolic requirements, while wombats with early signs of mange (highest segment score of greater than or equal to 3, *n* = 2) would require 602 g d^−1^ (±80.43 g). This equates to 33.2% greater food requirement for animals with signs of early-stage mange compared with healthy individuals.

### 3.3. Aim III: mange-induced changes in foraging and inactivity

**Figure 5.**
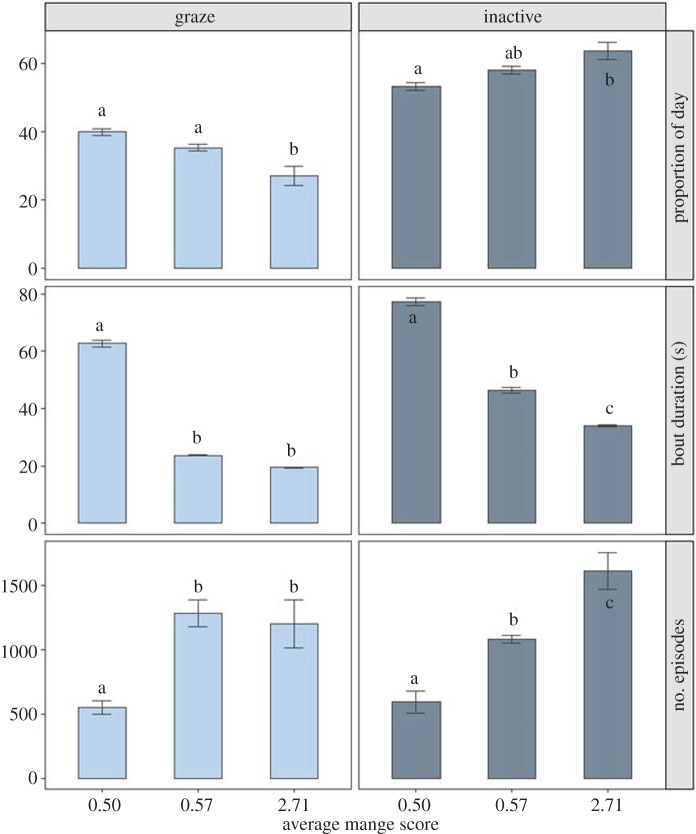
Mange-induced changes in bare-nosed wombat grazing and inactivity behaviours. Three wombats, one healthy (mange score 0.5), one with ambiguous signs of early mange (mange score 0.57) and one with moderate mange (mange score 2.71), were observed for grazing and inactivity behaviours across four full circadian cycles (days). Each day was composed of 28 800 3 s epochs, and each epoch was assigned to the activity that lasted the majority of the 3 s, giving a total of 345 600 epochs manually scored. Behavioural changes were analysed using three types of data: average number of episodes per activity per 24 h day, average bout duration per activity and proportion (%) of day spent engaging in each activity. Significant differences between values are indicated by labels ‘a’, ‘b’ and ‘c’.

The mange-infected wombat spent significantly less time grazing during the 24 h day than healthier animals (*F*_2,6_ = 13.33, *p* < 0.01; healthy, 39.8 ± 1.0% s.e.; early, 35.2 ± 1.0% s.e.; moderate, 27.0 ± 2.7% s.e.) (figure 5). This reduction in grazing arose from shorter grazing bouts that were one-third the duration as that observed in the healthiest animal (*F*_2,12133_ = 208.6, *p* < 0.01; healthy, 62.6 ± 1.3 s s.e.; early 23.7 ± 0.2 s s.e.; moderate, 19.4 ± 0.1 s s.e.). Those with the highest mange scores engaged in more grazing episodes than the wombat with the lowest mange score (*F*_2,6_ = 18.89, *p* < 0.01; healthy, 549.5 ± 53.7 s.e.; early, 1284.3 ± 103.7 s.e., moderate, 1201.0 ± 186.1 s.e.). Conversely, the amount of inactivity increased with the average mange score (*F*_2,6_ = 8.85, *p* = 0.02; healthy, 53.1 ± 1.2% s.e.; early, 57.8 ± 1.8% s.e.; moderate, 63.5 ± 2.6% s.e.). This pattern was mirrored by an increase in the number of episodes of inactivity, indicating that more diseased animals had more episodes of inactivity (*F*_2,6_ = 31.19, *p* < 0.01; healthy, 594.5 ± 87.0 s.e.; early, 1081.3 ± 30.4 s.e.; moderate, 1613.8 ± 142.4 s.e.), yet these episodes were of a shorter duration (*F*_2,13152_ = 67.10, *p* < 0.01; healthy, 77.2 ± 1.5 s s.e.; early, 46.2 ± 1.0 s s.e.; moderate, 34.0 ± 0.3 s s.e.). The individual with the highest mange score spent the most amount of time scratching (electronic supplementary material, D and E).

Realized feeding rates differed between healthy and mange-infected wombats. The healthy wombat spent about 39% of the day foraging, while the mangy wombat only spent about 27% of the day foraging (using data from W006, only) (electronic supplementary material, E). Additionally, healthy wombats have bite rates of 84 bites min^−1^, and mange-infected wombats have an estimated bite rate of 71 bites min^−1^ [19]. With these foraging and bite rates, healthy wombats are able to consume 748.2 g of plant matter per day (equivalent to 5536 kJ d^−1^), which is sufficient to meet or exceed their daily energy requirements (3344.9 ± 226 kJ d^−1^ s.e.; table 1). Conversely, mange-infected wombats can ingest approximately 439.5 g of plant matter per day (equivalent to 3252 kJ d^−1^), falling short of their daily needs (4457.4 ± 595.2 kJ d^−1^ s.e.; table 1).

### 3.4. Aim IV: fatty acid composition

A total of 34 fatty acids were incorporated into a PCA, as well as fat sums (e.g. total monounsaturated, total polyunsaturated) (electronic supplementary material, G). The PCA results revealed 14 fatty acids to have the strongest explanatory power: palmitic, stearic, oleic, eicosenoic, omega 6 (*n*-6), omega 3 (*n*-3), *n*-6 linoleic, *n*-3 alpha-linolenic, *n*-6 arachidonic, *n*-6 docosatetraenoic, *n*-3 docosahexaenoic, total polyunsaturated, total saturated and total monounsaturated. The relationship between average mange scores and the proportion of these 14 fatty acids present in each tissue type are represented visually (electronic supplementary material, H).

**Figure 6.**
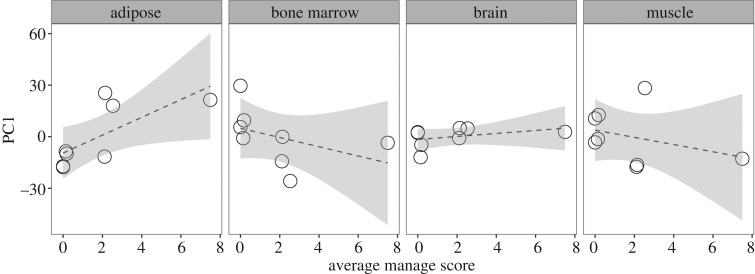
The relationship between average mange severity score and the fatty acid composition of four tissue types. Regressions were run for each tissue type using PC1 predictor values (from PCA of 14 fatty acids, table 2) and average mange severity scores. There was a significant relationship between adipose fatty acid composition and mange severity (*F*_1,6_ = 6.40, *p* = 0.04, *R*^2^ = 0.44), but no significant relationship in other tissues (bone marrow *F*_1,6_ = 1.25, *p* = 0.30, *R*^2^ = 0.03; brain *F*_1,6_ = 1.04, *p* = 0.35, *R*^2^ = 0.01; muscle *F*_1,6_ = 0.73, *p* = 0.43, *R*^2^ = −0.04).

These 14 fatty acids were then used in a more restricted PCA (table 2), for which PC1 was evaluated as a linear response variable to wombat mange severity score for each tissue type (figure 6). There was a significant relationship between adipose fatty acid composition and mange severity (*F*_1,6_ = 6.40, *p* = 0.04, *R*^2^ = 0.44), but no relationship between fatty acid composition and mange severity in the bone marrow, brain and muscle tissues (*F*_1,6_ = 1.25, *p* = 0.30, *R*^2^ = 0.03; *F*_1,6_ = 1.04, *p* = 0.35, *R*^2^ = 0.01; *F*_1,6_ = 0.73, *p* = 0.43, *R*^2^ = −0.04, respectively). The adipose fatty acid results suggest that as mange severity increases, omega-6 and arachidonic acid (C20 : 4) increase, and oleic acid (C18 : 1), alpha linoleic acid (C18 : 3) and total monounsaturated fats decrease (table 2, figure 6).

**Table 2.** PCA results from fat composition in four wombat tissues. (PC1 loading values are presented for 11 individual fatty acids and three acid type summations (total polyunsaturated, total saturated, total monounsaturated). PC1 loading values ≥ 0.3 in italics.)

		adipose	bone marrow	brain	muscle
fatty acid	type^a^	83.8%	55.4%	86.8%	75.1%
C16 : 0 palmitic	SFA	−0.19	*0*.*31*	−0.17	0.22
C18 : 0 stearic	SFA	0.24	−*0*.*31*	0.02	0.02
C18 : 1 oleic	MUFA	−*0*.*31*	*0*.*35*	0.29	0.16
C20 : 1 eicosenoic	MUFA	0.01	−0.01	*0*.*33*	0.00
omega-6	PUFA	*0*.*55*	−*0*.*43*	−0.05	−*0*.*56*
C18 : 2 *n*-6 linoleic	PUFA	0.12	−0.10	−0.11	−*0*.*52*
C20 : 4 *n*-6 arachidonic	PUFA	*0*.*30*	−0.14	0.02	−0.04
C22 : 4 *n*-6 docosatetraenoic	PUFA	0.04	−0.02	0.04	0.00
omega-3	PUFA	−0.28	−0.09	−*0*.*35*	0.09
C18 : 3 *n*-3 alpha-linolenic	PUFA	−*0*.*39*	0.06	−0.24	0.11
C22 : 6 *n*-3 docosahexaenoic	PUFA	0.02	−0.02	−0.07	−0.01
total polyunsaturated	**—**	0.27	−*0*.*51*	−*0*.*40*	−*0*.*47*
total saturated	—	0.04	0.07	−0.21	0.26
total monounsaturated	—	−*0*.*32*	*0*.*44*	*0*.*60*	0.19

^a^SFA, saturated fatty acid; MUFA, monounsaturated fatty acid; PUFA, polyunsaturated fatty acid.

## 4. Discussion

The term ‘disease’ is a manifestation of all the consequences of infection. The impact of infection can be diverse, direct or indirect, difficult to measure and can have cascading interactions resulting in amplified effects. Sarcoptic mange is an emerging infectious disease of mammals [8], affecting more than 100 species worldwide [9], with generally conserved effects across species. Thus, addressing the individual and compounding effects of mange-induced changes in one host may have implications for other host species. Here, we bridge critical knowledge gaps regarding the impacts of physiological changes on host metabolism, thermal energetic demands, effects on host behaviour and fat composition. Specifically, we show that mange-infected wombats experience heightened energetic demands through heat loss and raised metabolism. We find that wombats cannot compensate for the increased metabolic requirement through altering foraging behaviours (indeed they spend more time inactive), and subsequently deplete their fat stores, with altered fatty acid composition in adipose tissues, but not necessarily other tissues. These findings improve our understanding of the process by which *S. scabiei* infection results in host physiological changes, progressive disease phenotypes and mortality; and, may also contribute to other globally important chronic inflammatory parasitic infections of animals, such as notoedric mange [54].

In mammals, hair plays a major role in the conservation of energy and regulation of the daily energy budget. However, disruption of the pelt-environment interface by means of alopecia (e.g. from *S. scabiei* infection) can result in inefficient thermoregulation and excessive heat loss to the environment through the skin. This can be particularly impactful when alopecia is substantial (greater than 50%) across the host body. Alopecia with subsequent heat loss is observed in a range of parasite systems (e.g. ticks on moose [55]; *S. scabiei* in wolves [18]; *Demodex* spp. mites in mule deer [56]), with implications for increased energetic burden on the host. We found that infected wombats can lose between approximately 140 and 235 kJ h^−1^ (1.1–1.8 MJ d^−1^, assuming 8 h of activity) through their alopecia-impacted FL, St and HL, while healthy wombats lose as little as approximately 40–90 kJ h^−1^ (0.3–0.7 MJ d^−1^). This translates to 1.56–5.88 times more energy loss. Additionally, this energy burden may be much higher (per day) in mange-infected wombats, owing to their tendency to spend more time outside of the burrow [19], and thus experience increased conductive heat loss to flowing air. However, this potential increased cost may be ameliorated through their shift towards diurnal activity [24]. Despite the likely underestimation of heat loss in mange-infected wombats owing to the use of only three body segments, these rates are comparable with those of small wolves (around twice the mass of wombats) with early mange, which lose approximately 3.5–6.5 MJ per night [18]. These findings provide insight into the energetic cost of alopecia and heat loss in mange-infected wombats, and suggest that heat loss may play a major role in changes to metabolism (see below).

We found that the compounding impacts of host responses to *S. scabiei* infection have metabolic consequences that the host cannot sustain long term. There is broad consensus that prompting an immune response is energetically expensive [57,58], and this energetic burden can also be exacerbated through physiological changes. Wombats infected with mange experienced a 40% increase in their field metabolic rate compared with that of healthy wombats (155.4 kJ kg^−1^ d^−1^, 218.3 kJ kg^−1^ d^−1^, respectively), a rate that would require infected individuals to consume on average approximately 150 g more of plant matter per day to meet their metabolic needs. While these field metabolic rates fall within the documented range for mainland bare-nosed wombats during the wet season [51], the baseline metabolic requirements for individuals living in Tasmania are likely to be lower than those from mainland Australia, owing to cooler seasonal daily temperatures [59]. Survival of mange-infected wombats with increased energetic demands will depend on their ability to increase their energy intake and may require behavioural plasticity.

The energetic and physiological effects of disease presence can also induce host behavioural changes [19,57,60,61], through direct or indirect impacts. For example, a host impacted by disease may adjust behaviours owing to direct impacts, such as reduced mobility or function, or owing to indirect impacts, such as engaging in new activities in response to the disease that necessarily decrease the time available for other behaviours. Our findings add to previous research and suggest that *S. scabiei*-infected wombats attempt to increase their foraging effort to compensate for the energetic demands of mange; but, here we show why they are unsuccessful in doing so.

Mange-infected wombats engage in more periods of foraging behaviour than healthy wombats, but are unable to sustain foraging efforts for extended periods of time, resulting in a smaller proportion of the day spent foraging, overall. Additionally, mangy wombats are unable to engage in extended periods of rest, probably owing to the epidermal irritation caused by the mite [1,2]. Mange-infected wombats engage in more periods of inactivity similar to foraging, but unlike the decrease in foraging observed in mangy wombats, inactivity increased in those with the highest mange score. The inability for diseased wombats to maintain periods of both foraging and inactivity may be owing to the interruption by mange-related activities, such as scratching. Indeed, wombats with more severe mite infestations spend more time scratching than healthier animals [19,35]. Combined, our results suggest that when the energetic pressure of mange is too high, the host may not be able to compensate. In this case, the daily energy intake required to survive exceeds the metabolizable daily energy intake rate. Furthermore, shorter and interrupted periods of inactivity may reduce their ability to conserve energy by resting (as seen in other species; [39]). It is important to note that these conclusions have been derived from a modest sample size, and further investigation into mange-induced host behavioural changes would greatly improve our understanding of the disease.

We were also motivated to understand if *S. scabiei* infection alters fat composition across tissues, and thus, impacts functions that could be connected to other aspects of mange disease (e.g. behaviour). When hosts cannot ameliorate energetic demands of disease, they must draw on energy stores to survive. The most obvious consequence of this is emaciation, with less obvious impacts on fat composition in vital tissues, which feed back into the cascade of disease impacts. We found that effects of *S. scabiei* infection on fat composition were most obvious in adipose tissues, where increased levels of *n*-6 arachidonic acid and decreased levels of countering *n*-3 fatty acids (alpha-linolenic) were observed in mange-infected wombats. *n*-6 acids, specifically arachidonic acid, promote a range of physiological effects, including inflammation, arrhythmia, platelet activation and vasoconstriction [62]. *n*-3 fatty acids can counter the effects of *n*-6 acids; however, a high *n*-6 : *n*-3 ratio results in an inflammatory signalling response [62]. Additionally, oleic acid, a fatty acid that plays a role in anti-inflammatory response, activation of immune cells and cutaneous wound repair, decreases as mange severity increases [63,64]. Thus, our fatty acid results are indicative of functional shifts towards generalized chronic inflammatory, and inhibited anti-inflammatory, functional responses in wombats.

The fatty acid results provide another line of evidence supporting a primary immune response to *S. scabiei* of inflammation in an attempt to clear the parasite and repair tissues [1]. However, prolonged inflammation without mediation has a metabolic cost, and can result in loss of tissue function [65]. Skin lesions are also common signs of mange disease [1], and the inability to heal lesions makes the host prone to secondary bacterial infections [66]. The imbalance in fatty acid composition in mange-infected wombats probably contributes to the progression of the physiological effects of mange disease, and may accelerate host mortality. Further research into the consequences of prolonged inflammation, even after the infection has been cleared, will be critical for wildlife recovery.

This study contributes to the rich body of knowledge linking *S. scabiei* infection to the phenotype of mange disease. We establish new connections showing that: (i) mangy wombats lose a greater amount of heat to the environment, with substantial energetic cost, (ii) mange infection causes an increase in field metabolic rate that requires increased foraging activity to counter, (iii) infected wombats cannot effectively meet this increased metabolic demand through increased foraging efforts and actually increase their time spent inactive and, (iv) mange infection results in an imbalance of fatty acids, which may feedback into the cascade of physiological impacts of disease, particularly associated with chronic inflammation. Sarcoptic mange is an emerging infectious disease that causes significant disease burden, economic impacts in animal production industries, and has raised conservation concerns in wildlife populations [8,9,67]. Our research has implications for the treatment and rehabilitation of mange-infected individuals. For example, it may be practical to combine treatment efforts with high calorie food supplementation to efficiently combat mange infection in wild and domestic animals. Such an approach may also be possible at population scales, and may enhance the outcomes of treatment methods in the field. Further research in this area would be valuable.

## Supplementary Material

Mange severity score chart

## Supplementary Material

Wombat location details

## Supplementary Material

Equations for sensible heat loss calculation

## Supplementary Material

Wombat activity breakdown

## Supplementary Material

Wombat circadian behaviour

## Supplementary Material

Wombat mange and fatty acid composition.

## Data accessibility

All data are accessible through Dryad (http://dx.doi.org/10.5061/dryad.061v7) [68] and the electronic supplementary material.
