# Supplementary material for: The cascading pathogenic consequences of Sarcoptes scabiei infection that manifest in host disease
Source: R Soc Open Sci. 2018 Apr 18;5(4):180018. doi: 10.1098/rsos.180018 (PMC5936957; doi:10.1098/rsos.180018)
Supplement: Mange severity score chart [file rsos180018supp1.docx]

Supplementary Material A. Mange severity score chart.

A. Mange scores based on hair loss percentage.

| *Score* | *Hair loss* |
| --- | --- |
| 0 | No signs, healthy wombat |
| 1 | Ambiguous; possible hair thinning, but not clear |
| 2 | Clear signs of hair thinning, possible skin reddening |
| 3 | Small bald patches ≤10% of area |
| 4 | Moderate bald patches 10-20% |
| 5 | 20-30% |
| 6 | 30-40% |
| 7 | 40-50% |
| 8 | 50-60% |
| 9 | 60-70% |
| 10 | ≥70% of body covered, very poor body condition, severe |
