# Supplementary material for: The cascading pathogenic consequences of Sarcoptes scabiei infection that manifest in host disease
Source: R Soc Open Sci. 2018 Apr 18;5(4):180018. doi: 10.1098/rsos.180018 (PMC5936957; doi:10.1098/rsos.180018)
Supplement: Wombat location details [file rsos180018supp2.docx]

Supplementary Material B. Wombat location details.

B. Individual wombat details used for Aims I, III, and IV. Location Grid zone designation is 55G.

| *Aim I - Quantifying heat loss* | | | | | | |
| --- | --- | --- | --- | --- | --- | --- |
| Wombat ID | Age | Sex | Season | Location | UTM1 | UTM2 |
| T01 | J | - | Mar. 2014 | Narawntapu | 0466482 E | 5444789 N |
| T02a | A | - | Mar. 2014 | Narawntapu | 0466482 E | 5444789 N |
| T02b | A | - | Mar. 2014 | Narawntapu | 0466482 E | 5444789 N |
| T10 | A | - | Apr. 2014 | Narawntapu | 0466482 E | 5444789 N |
| T14 | A | - | June 2014 | Narawntapu | 0466482 E | 5444789 N |
| *Aim III - Resting and foraging behaviour* | | | | | | |
| Wombat ID | Age | Sex | Season | Location | UTM1 | UTM2 |
| W002 | A | F | Apr. / May2015 | Narawntapu | 0466482 E | 5444789 N |
| W006 | A | F | Apr. / May2015 | Narawntapu | 0466482 E | 5444789 N |
| W009 | A | M | Apr. / May2015 | Narawntapu | 0466482 E | 5444789 N |
| *Aim IV - Fat composition* | | | | | | |
| Wombat ID | Age | Sex | Season | Location | UTM1 | UTM2 |
| F02 | A | M | Sept. 2015 | Port Arthur | 0567965 E | 5223099 N |
| F03 | A | M | Dec. 2015 | Forcett | - | - |
| F04 | A | F | Dec. 2015 | Anthill Ponds | - | - |
| F05 | A | F | Apr. 2016 | Broadmarsh | 0510025 E | 5276659 N |
| F08 | A | M | Feb. 2016 | East Derwent | - | - |
| F09 | A | F | June 2016 | Tasmania | - | - |
| F10 | A | M | July 2015 | Brighton | 0520463 E | 5272433 N |
| F11 | A | F | Aug. 2015 | Brighton | - | - |
