# Supplementary material for: The cascading pathogenic consequences of Sarcoptes scabiei infection that manifest in host disease
Source: R Soc Open Sci. 2018 Apr 18;5(4):180018. doi: 10.1098/rsos.180018 (PMC5936957; doi:10.1098/rsos.180018)
Supplement: Equations for sensible heat loss calculation [file rsos180018supp3.docx]

Supplementary Material C. Equations for sensible heat loss calculation.

C. Sensible heat loss was defined as the sum of convective heat loss (free and forced) and radiative heat loss, following the methods of Cross *et al*. (1). Free and forced convective heat losses were calculated using the following equations:

Eq. 1 $Nu free=B {Gr}^{m}$

Eq. 2 $Nu forced=D {Re}^{n}$

where *Gr* is the Grashof number, *Re* is the Renolds number, and *B*, *m*, *D*, and *e* are constants based on surface shape and orientation (2). Radiative heat loss was calculated using the equation:

Eq. 3 $R= \frac{1}{A}\intԐ \sigma\left( T_{surface}^{4}- T_{surrounding}^{4} \right) d A$

whereby *A* is the area of the body region, Ԑ is the emissivity of the wombat fur (assumed to be 0.95), *T* _surface_ is the temperature of the wombat segment derived from the thermal imagery, and *T* _surrounding_ is the ambient temperature.

References

1. Cross PC*, et al.* (2016) Energetic costs of mange in wolves estimated from infrared thermography. *Ecology* 97(8):1938-1948.

2. Monteith J & Unsworth M (2013) *Principles of environmental physics: plants, animals, and the atmosphere* (Academic Press (Elsevier), Oxford, UK) 4th Ed.
