# Supplementary material for: The cascading pathogenic consequences of Sarcoptes scabiei infection that manifest in host disease
Source: R Soc Open Sci. 2018 Apr 18;5(4):180018. doi: 10.1098/rsos.180018 (PMC5936957; doi:10.1098/rsos.180018)
Supplement: Wombat activity breakdown [file rsos180018supp4.docx]

Supplementary Material D and E. Wombat activity breakdown

D. Breakdown of behaviours for each wombat (W002, W009, and W006) across four days.

| W002 - Healthy (M=1) | | | | | | | | | | | | |
| --- | --- | --- | --- | --- | --- | --- | --- | --- | --- | --- | --- | --- |
| Activity | **Day 1 (April 24)** | | | **Day 2 (April 27)** | | | **Day 3 (April 30)** | | | **Day 4 (May 4)** | | |
|  | Total # of bouts | Avg. bout dur. (sec) | Longest bout (sec) | Total # of bouts | Avg. bout dur. (sec) | Longest bout (sec) | Total # of bouts | Avg. bout dur. (sec) | Longest bout (sec) | Total # of bouts | Avg. bout dur. (sec) | Longest bout (sec) |
| Inactive | 489 | 93.28 | 1563 | 774 | 60.62 | 2175 | 707 | 60.98 | 2202 | 408 | 117.46 | 2136 |
| Restlessness | 180 | 11.97 | 102 | 274 | 15.30 | 438 | 315 | 14.62 | 315 | 58 | 12.05 | 81 |
| Slow walk / graze | 422 | 85.11 | 2802 | 657 | 48.98 | 1239 | 617 | 55.45 | 2133 | 502 | 70.39 | 1350 |
| Steady Walk | 131 | 11.68 | 81 | 118 | 15.43 | 108 | 199 | 12.99 | 78 | 96 | 11.91 | 75 |
| Dig | 62 | 3.68 | 9 | 93 | 3.41 | 9 | 141 | 3.42 | 12 | 130 | 3.39 | 9 |
| Scratch | 55 | 4.15 | 12 | 44 | 4.16 | 9 | 56 | 4.18 | 9 | 31 | 4.74 | 12 |
| Run | 7 | 4.71 | 9 | 3 | 4.00 | 6 | 3 | 5.00 | 6 | 3 | 4.00 | 6 |
| Unknown 1 | 45 | 4.60 | 21 | 38 | 4.66 | 12 | 70 | 5.31 | 18 | 39 | 4.69 | 15 |
| Unknown 2 | 49 | 5.88 | 15 | 66 | 5.59 | 15 | 102 | 4.88 | 15 | 49 | 5.33 | 15 |
| Unknown 3 | 5 | 3.00 | 3 | 2 | 4.50 | 6 | 16 | 3.19 | 6 | 5 | 3.00 | 3 |
| Unknown 4 | 54 | 3.44 | 9 | 56 | 3.59 | 9 | 56 | 3.80 | 12 | 61 | 3.64 | 6 |

| W009 – Early (Ambiguous) (M=2) | | | | | | | | | | | | |
| --- | --- | --- | --- | --- | --- | --- | --- | --- | --- | --- | --- | --- |
| Activity | **Day 1 (April 24)** | | | **Day 2 (April 27)** | | | **Day 3 (April 30)** | | | **Day 4 (May 4)** | | |
|  | Total # of bouts | Avg. bout dur. (sec) | Longest bout (sec) | Total # of bouts | Avg. bout dur. (sec) | Longest bout (sec) | Total # of bouts | Avg. bout dur. (sec) | Longest bout (sec) | Total # of bouts | Avg. bout dur. (sec) | Longest bout (sec) |
| Inactive | 1061 | 44.59 | 2400 | 1087 | 46.79 | 2079 | 1016 | 50.80 | 3084 | 1161 | 43.12 | 3054 |
| Restlessness | 201 | 14.33 | 144 | 110 | 16.15 | 744 | 120 | 21.83 | 1089 | 54 | 17.11 | 114 |
| Slow walk / graze | 1075 | 30.27 | 516 | 1246 | 24.47 | 531 | 1245 | 22.66 | 618 | 1571 | 19.39 | 387 |
| Steady Walk | 180 | 11.60 | 72 | 141 | 11.09 | 66 | 129 | 13.50 | 84 | 168 | 11.59 | 66 |
| Dig | 184 | 3.24 | 12 | 270 | 3.90 | 15 | 384 | 3.90 | 18 | 531 | 4.53 | 18 |
| Scratch | 42 | 3.86 | 15 | 10 | 3.30 | 6 | 15 | 4.80 | 9 | 17 | 3.88 | 6 |
| Run | 15 | 14.00 | 39 | 5 | 10.80 | 36 | 0 | 0.00 | 0 | 2 | 4.50 | 6 |
| Unknown 1 | 40 | 6.15 | 21 | 39 | 4.92 | 18 | 47 | 6.26 | 21 | 38 | 4.97 | 12 |
| Unknown 2 | 47 | 5.49 | 18 | 60 | 4.65 | 12 | 45 | 6.20 | 18 | 44 | 5.93 | 18 |
| Unknown 3 | 3 | 3.00 | 3 | 3 | 4.00 | 6 | 2 | 4.50 | 6 | 2 | 3.00 | 3 |
| Unknown 4 | 26 | 3.69 | 6 | 20 | 3.90 | 6 | 15 | 3.40 | 9 | 14 | 3.86 | 12 |

| W006 - Moderate (M=6) | | | | | | | | | | | | |
| --- | --- | --- | --- | --- | --- | --- | --- | --- | --- | --- | --- | --- |
| Activity | **Day 1 (April 24)** | | | **Day 2 (April 27)** | | | **Day 3 (April 30)** | | | **Day 4 (May 4)** | | |
|  | Total # of bouts | Avg. bout dur. (sec) | Longest bout (sec) | Total # of bouts | Avg. bout dur. (sec) | Longest bout (sec) | Total # of bouts | Avg. bout dur. (sec) | Longest bout (sec) | Total # of bouts | Avg. bout dur. (sec) | Longest bout (sec) |
| Inactive | 1220 | 49.56 | 750 | 1821 | 28.68 | 738 | 1589 | 35.29 | 1191 | 1825 | 27.72 | 735 |
| Restless | 364 | 15.57 | 408 | 327 | 11.30 | 108 | 115 | 11.53 | 114 | 168 | 8.09 | 108 |
| Slow walk / graze | 652 | 26.12 | 198 | 1376 | 19.22 | 432 | 1306 | 17.13 | 225 | 1470 | 18.67 | 246 |
| Steady Walk | 2 | 22.50 | 36 | 42 | 14.65 | 45 | 114 | 19.98 | 90 | 110 | 21.19 | 81 |
| Dig | 15 | 4.40 | 12 | 10 | 4.64 | 9 | 12 | 3.69 | 12 | 18 | 5.68 | 12 |
| Scratch | 65 | 3.05 | 6 | 96 | 3.09 | 9 | 63 | 3.10 | 6 | 114 | 3.34 | 18 |
| Run | 0 | 0.00 | 0 | 0 | 0.00 | 0 | 0 | 0.00 | 0 | 1 | 6.00 | 6 |
| Unknown 1 | 165 | 6.24 | 27 | 147 | 8.55 | 39 | 195 | 7.85 | 27 | 186 | 9.56 | 60 |
| Unknown 2 | 186 | 8.53 | 30 | 173 | 9.05 | 42 | 273 | 8.46 | 54 | 235 | 8.85 | 45 |
| Unknown 3 | 1 | 6.00 | 6 | 1 | 3.00 | 3 | 1 | 0.00 | 0 | 1 | 6.00 | 6 |
| Unknown 4 | 81 | 3.74 | 12 | 64 | 3.61 | 6 | 72 | 3.50 | 6 | 84 | 3.43 | 9 |

| W002 - Healthy (M=1) | | | | | | | | | | |
| --- | --- | --- | --- | --- | --- | --- | --- | --- | --- | --- |
| Activity | Number of Bouts (all days) | | | | Bout Duration (sec) (all days) | | | | Average time spent (per day) | |
|  | Average | S.D. | S.E. | | Average | | S.D. | S.E. | % | S.E. |
| Inactive | 594.5 | 173.96 | 86.98 | | 77.20 | | 73.30 | 1.50 | 53.12 | 1.20 |
| Restless | 206.75 | 114.14 | 57.07 | | 14.09 | | 11.01 | 0.38 | 3.37 | 1.06 |
| Slow Wlk. grz. | 549.5 | 107.43 | 53.72 | | 62.62 | | 62.11 | 1.32 | 39.83 | 0.95 |
| Stdy. wlk. | 136 | 44.41 | 22.21 | | 13.09 | | 4.26 | 0.18 | 2.06 | 0.35 |
| Dig | 106.5 | 36.08 | 18.04 | | 3.47 | | 0.42 | 0.02 | 0.43 | 0.07 |
| Scratch | 46.5 | 11.68 | 5.84 | | 4.26 | | 0.69 | 0.05 | 0.23 | 0.02 |
| Run | 4 | 2.00 | 1.00 | | 4.50 | | 0.63 | 0.16 | 0.02 | 0.01 |
| Unk. 1 | 48 | 14.99 | 7.49 | | 4.89 | | 0.99 | 0.07 | 0.27 | 0.05 |
| Unk. 2 | 66.5 | 24.99 | 12.49 | | 5.32 | | 0.98 | 0.06 | 0.41 | 0.06 |
| Unk. 3 | 7 | 6.16 | 3.08 | | 3.21 | | 0.26 | 0.05 | 0.03 | 0.01 |
| Unk. 4 | 56.75 | 2.99 | 1.49 | | 3.62 | | 0.49 | 0.03 | 0.24 | 0.01 |
| W009 - Early (M=2) | | | | | | | | | | |
| Activity | Number of Bouts (all days) | | | | | Bout Duration (sec) (all days) | | | Average time spent (per day) | |
|  | Average | S.D. | | S.E. | | Average | S.D. | S.E. | % | S.E. |
| Inactive | 1081.25 | 60.72 | | 30.36 | | 46.21 | 65.33 | 0.99 | 57.82 | 1.08 |
| Restless | 121.25 | 60.58 | | 30.29 | | 16.91 | 21.46 | 0.97 | 2.37 | 0.51 |
| Slow Wlk. grz. | 1284.25 | 207.38 | | 103.69 | | 23.69 | 13.70 | 0.19 | 35.21 | 1.02 |
| Stdy. wlk. | 154.5 | 23.56 | | 11.78 | | 11.94 | 3.84 | 0.15 | 2.13 | 0.13 |
| Dig | 342.25 | 150.15 | | 75.07 | | 4.07 | 0.70 | 0.02 | 1.61 | 0.45 |
| Scratch | 21 | 14.31 | | 7.15 | | 3.96 | 0.71 | 0.08 | 0.10 | 0.03 |
| Run | 5.5 | 6.66 | | 3.33 | | 11.87 | 3.65 | 0.78 | 0.08 | 0.06 |
| Unk. 1 | 41 | 4.08 | | 2.04 | | 5.62 | 1.11 | 0.09 | 0.27 | 0.03 |
| Unk. 2 | 49 | 7.44 | | 3.72 | | 5.49 | 1.07 | 0.08 | 0.31 | 0.01 |
| Unk. 3 | 2.5 | 0.58 | | 0.29 | | 3.60 | 0.42 | 0.13 | 0.01 | 0.00 |
| Unk. 4 | 18.75 | 5.50 | | 2.75 | | 3.72 | 0.54 | 0.06 | 0.08 | 0.01 |
| W006 - Moderate (M=6) | | | | | | | | | | |
| Activity | Number of Bouts (all days) | | | | | Bout Duration (sec) (all days) | | | Average time spent (per day) | |
|  | Average | S.D. | | S.E. | | Average | S.D. | S.E. | % | S.E. |
| Inactive | 1613.75 | 284.74 | | 142.37 | | 33.98 | 25.56 | 0.32 | 63.47 | 2.55 |
| Restless | 243.5 | 120.70 | | 60.35 | | 12.37 | 9.28 | 0.30 | 3.49 | 1.21 |
| Slow Wlk. grz. | 1201 | 372.12 | | 186.06 | | 19.42 | 8.68 | 0.13 | 27.00 | 2.74 |
| Stdy. wlk. | 67 | 54.49 | | 27.25 | | 19.87 | 5.56 | 0.34 | 1.54 | 0.68 |
| Dig | 13.75 | 3.50 | | 1.75 | | 4.96 | 0.95 | 0.13 | 0.08 | 0.02 |
| Scratch | 84.5 | 24.80 | | 12.40 | | 3.17 | 0.37 | 0.02 | 0.31 | 0.05 |
| Run | 0.25 | 0.50 | | 0.25 | | 1.50 | 1.00 | 1.00 | 0.00 | 0.00 |
| Unk. 1 | 173.25 | 21.55 | | 10.77 | | 8.07 | 1.99 | 0.08 | 1.62 | 0.19 |
| Unk. 2 | 216.75 | 46.03 | | 23.02 | | 8.70 | 1.79 | 0.06 | 2.18 | 0.21 |
| Unk. 3 | 1 | 0.00 | | 0.00 | | 3.75 | 0.96 | 0.48 | 0.00 | 0.00 |
| Unk. 4 | 75.25 | 9.07 | | 4.53 | | 3.57 | 0.45 | 0.03 | 0.31 | 0.02 |

E. Behaviours averaged across four days for each wombat.
