# Supplementary material for: The cascading pathogenic consequences of Sarcoptes scabiei infection that manifest in host disease
Source: R Soc Open Sci. 2018 Apr 18;5(4):180018. doi: 10.1098/rsos.180018 (PMC5936957; doi:10.1098/rsos.180018)

Supplementary Material F. Wombat circadian behaviour.

F. Circadian behaviour and force of activity for three wombats, with varying mange severities, across 16 days.


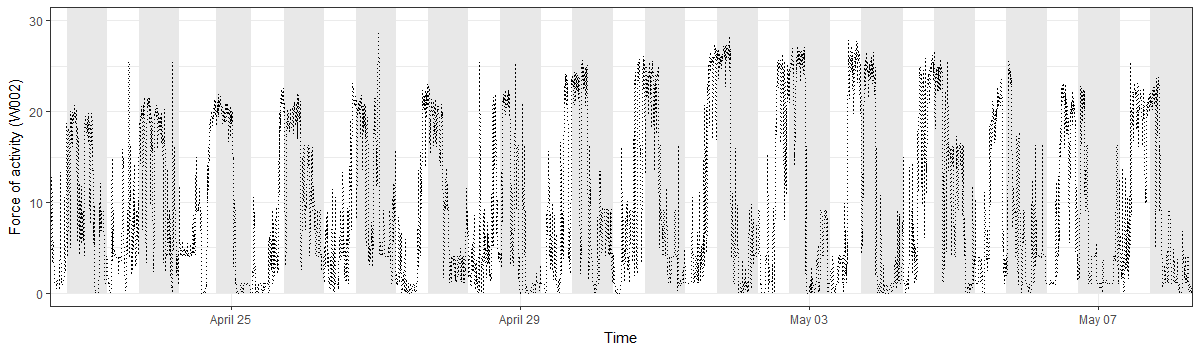

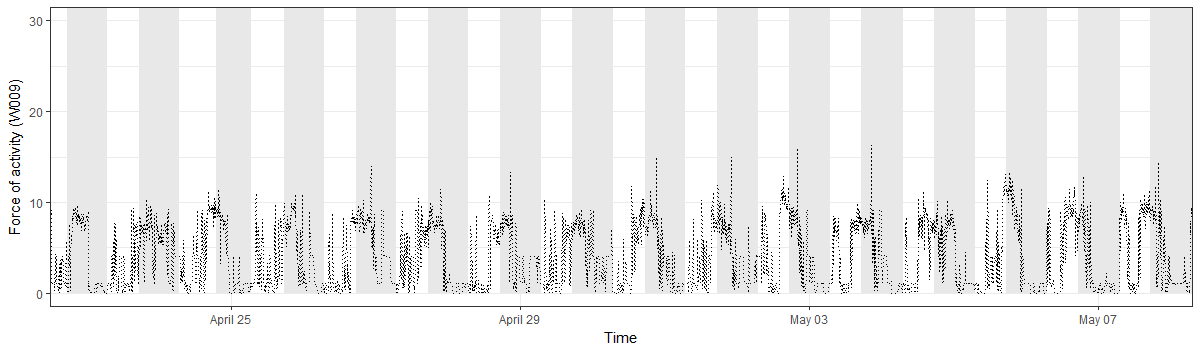

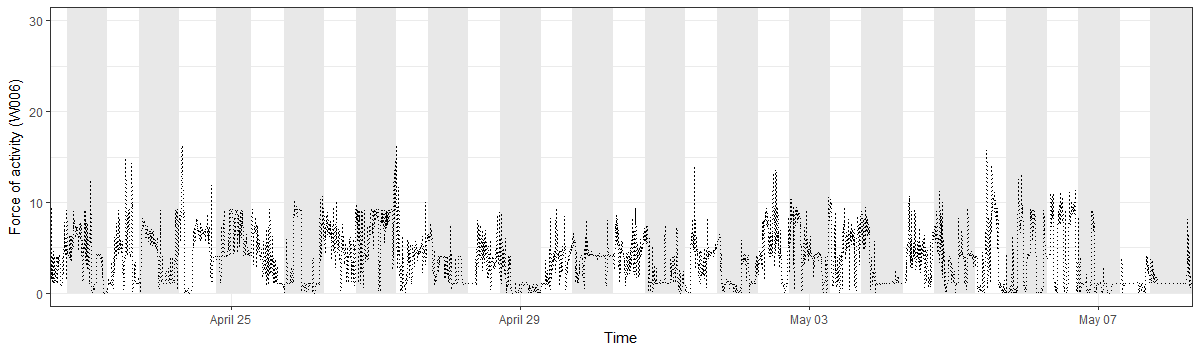

Supplement: Wombat circadian behaviour [file rsos180018supp5.docx]
