# Supplementary material for: The cascading pathogenic consequences of Sarcoptes scabiei infection that manifest in host disease
Source: R Soc Open Sci. 2018 Apr 18;5(4):180018. doi: 10.1098/rsos.180018 (PMC5936957; doi:10.1098/rsos.180018)
Supplement: Wombat mange and fatty acid composition. [file rsos180018supp6.docx]

Supplementary Material G and H. Wombat mange and fatty acid composition.

G. Results from the screening principle component analysis to identify fatty acids with the best explanatory power.

| Fatty Acid | Brain | | | Fat | | | Bone marrow | | | Muscle | | |
| --- | --- | --- | --- | --- | --- | --- | --- | --- | --- | --- | --- | --- |
|  | PC1 | PC2 | PC3 | PC1 | PC2 | PC3 | PC1 | PC2 | PC3 | PC1 | PC2 | PC3 |
|  | 86.4% | 7.2% | 3.3% | 83.8% | 12.6% | 2.7% | 55.8% | 32.7% | 7.7% | 74.5% | 19.7% | 4.1% |
| C4:0 Butyric | 0.00 | 0.00 | 0.00 | 0.00 | 0.00 | 0.00 | 0.00 | 0.00 | 0.00 | -0.01 | -0.01 | 0.00 |
| C6:0 Caproic | 0.00 | 0.00 | 0.00 | 0.00 | 0.00 | 0.00 | 0.00 | 0.00 | 0.00 | 0.00 | 0.00 | 0.00 |
| C8:0 Caprylic | 0.00 | 0.00 | 0.00 | 0.00 | 0.00 | 0.00 | 0.00 | 0.00 | 0.00 | 0.00 | 0.00 | 0.00 |
| C10:0 Capric | 0.00 | 0.00 | 0.00 | 0.00 | 0.00 | 0.00 | 0.00 | 0.00 | 0.00 | 0.00 | 0.00 | 0.00 |
| C12:0 Lauric | 0.00 | 0.00 | 0.00 | 0.00 | 0.00 | 0.00 | 0.00 | 0.00 | 0.00 | 0.00 | 0.00 | 0.00 |
| C14:0 Myristic | -0.03 | 0.04 | -0.01 | -0.03 | -0.01 | -0.02 | -0.05 | -0.02 | -0.11 | 0.01 | 0.03 | 0.02 |
| C15:0 Pentadecanoic | -0.01 | 0.02 | 0.01 | 0.00 | 0.00 | 0.02 | -0.01 | -0.01 | -0.03 | 0.00 | 0.00 | -0.01 |
| C16:0 Palmitic | -0.17 | -0.07 | 0.13 | -0.19 | -0.20 | **-0.37** | **-0.30** | -0.22 | **-0.40** | 0.22 | 0.20 | -0.25 |
| C17:0 Margaric | -0.02 | 0.02 | 0.02 | -0.01 | -0.01 | 0.00 | -0.01 | -0.02 | -0.04 | 0.01 | 0.00 | -0.02 |
| C18:0 Stearic | 0.02 | **0.36** | **-0.42** | 0.24 | -0.10 | -0.17 | **0.31** | -0.19 | **0.43** | 0.02 | **-0.34** | -0.29 |
| C20:0 Arachidic | 0.00 | 0.02 | 0.00 | 0.01 | 0.00 | 0.00 | 0.00 | 0.00 | 0.00 | 0.00 | 0.00 | -0.01 |
| C22:0 Behenic | 0.00 | 0.00 | 0.00 | 0.01 | 0.00 | 0.02 | 0.00 | 0.00 | 0.00 | 0.00 | 0.00 | 0.00 |
| C24:0 Lignoceric | -0.01 | -0.06 | 0.01 | 0.02 | 0.00 | 0.02 | 0.00 | 0.00 | 0.01 | 0.00 | 0.00 | 0.00 |
| Total saturated | -0.21 | 0.29 | -0.27 | 0.04 | **-0.32** | **-0.50** | -0.06 | **-0.44** | -0.15 | 0.26 | -0.13 | **-0.52** |
| C14:1 Myristoleic | 0.00 | 0.00 | 0.00 | 0.00 | 0.00 | 0.00 | 0.00 | 0.00 | 0.00 | 0.00 | 0.00 | 0.00 |
| C16:1 Palmitoleic | -0.03 | 0.06 | 0.02 | -0.05 | -0.02 | -0.05 | -0.11 | -0.04 | -0.13 | 0.02 | 0.05 | 0.03 |
| C17:1 Heptadecenoic | 0.00 | 0.00 | 0.00 | 0.00 | 0.00 | 0.00 | 0.00 | 0.00 | 0.00 | 0.00 | 0.00 | 0.00 |
| C18:1 Oleic | 0.29 | 0.03 | **0.48** | **-0.30** | -0.18 | **0.47** | **-0.34** | 0.16 | 0.29 | 0.16 | **0.37** | **0.33** |
| C20:1 Eicosenic | **0.33** | 0.04 | -0.24 | 0.01 | 0.00 | 0.02 | 0.01 | -0.02 | -0.02 | 0.00 | 0.00 | 0.01 |
| C22:1 Docosenoic | 0.00 | -0.03 | -0.02 | 0.01 | 0.00 | 0.02 | 0.00 | 0.00 | 0.01 | 0.00 | 0.00 | 0.00 |
| C24:1 Nervonic | 0.01 | 0.01 | 0.01 | 0.01 | -0.01 | 0.00 | 0.00 | 0.00 | 0.01 | 0.00 | 0.00 | -0.01 |
| Total monounsaturated | **0.60** | 0.11 | 0.25 | **-0.32** | -0.22 | **0.46** | **-0.44** | 0.10 | 0.16 | 0.19 | **0.42** | **0.34** |
| Omega-6 | -0.05 | **-0.53** | -0.21 | **0.55** | 0.07 | 0.20 | **0.42** | -0.17 | -0.11 | **-0.56** | 0.04 | -0.05 |
| Omega-3 | **-0.35** | 0.25 | **0.35** | -0.28 | **0.46** | -0.14 | 0.07 | **0.50** | -0.02 | 0.09 | **-0.31** | **0.33** |
| C18:2-6 Linoleic | -0.11 | 0.12 | 0.06 | 0.12 | 0.20 | 0.22 | 0.10 | -0.02 | **-0.51** | **-0.51** | **0.45** | -0.26 |
| C18:3-6 gamma Linolenic | 0.00 | 0.00 | 0.00 | 0.00 | 0.00 | 0.00 | 0.00 | 0.00 | 0.00 | 0.00 | 0.00 | 0.00 |
| C18:3-3 alpha Linolenic | -0.24 | **0.33** | 0.13 | **-0.39** | **0.47** | -0.20 | -0.08 | **0.51** | -0.25 | 0.11 | -0.08 | 0.25 |
| C20:2-6 Eicosadienoic | 0.00 | -0.03 | -0.05 | 0.01 | 0.01 | 0.01 | 0.01 | -0.01 | 0.00 | 0.00 | 0.00 | 0.01 |
| C20:3-6 Eicosatrienoic | 0.01 | -0.02 | 0.01 | 0.08 | -0.03 | -0.02 | 0.15 | -0.12 | 0.00 | -0.01 | -0.06 | 0.01 |
| C20:3-3 Eicosatrienoic | -0.02 | 0.01 | 0.01 | 0.00 | 0.02 | -0.01 | 0.01 | 0.01 | -0.04 | 0.01 | -0.02 | 0.02 |
| C20:4-6 Arachidonic | 0.02 | -0.28 | 0.00 | 0.29 | -0.09 | 0.01 | 0.14 | -0.06 | 0.20 | -0.04 | **-0.32** | 0.17 |
| C20:5-3 Eicosapentaenoic | 0.00 | 0.08 | -0.13 | 0.03 | -0.01 | -0.01 | 0.04 | -0.02 | 0.03 | 0.00 | -0.06 | -0.01 |
| C22:2-6 Docosadienoic | 0.00 | 0.00 | 0.00 | 0.00 | 0.00 | 0.00 | 0.00 | 0.00 | 0.00 | 0.00 | 0.00 | -0.01 |
| C22:4-6 Docosatetraenoic | 0.04 | **-0.30** | -0.24 | 0.04 | -0.02 | -0.01 | 0.02 | 0.03 | 0.19 | 0.00 | -0.02 | 0.02 |
| C22:5-3 Docosapentaenoic | -0.01 | 0.03 | 0.01 | 0.06 | -0.01 | 0.04 | 0.08 | -0.05 | 0.00 | -0.02 | -0.11 | 0.05 |
| C22:6-3 Docosahexaenoic | -0.07 | -0.19 | **0.32** | 0.02 | 0.00 | 0.01 | 0.01 | 0.05 | 0.25 | -0.01 | -0.03 | 0.02 |
| Total polyunsaturated | **-0.40** | -0.28 | 0.14 | 0.27 | **0.53** | 0.06 | **0.49** | **0.34** | -0.13 | **-0.47** | -0.27 | 0.28 |
| Total monotrans | -0.02 | -0.03 | 0.01 | 0.00 | 0.01 | -0.04 | 0.00 | 0.00 | -0.01 | 0.01 | 0.00 | -0.06 |
| Total polytrans | -0.01 | 0.01 | 0.00 | 0.00 | 0.00 | -0.01 | 0.00 | 0.00 | 0.00 | 0.00 | -0.01 | -0.02 |


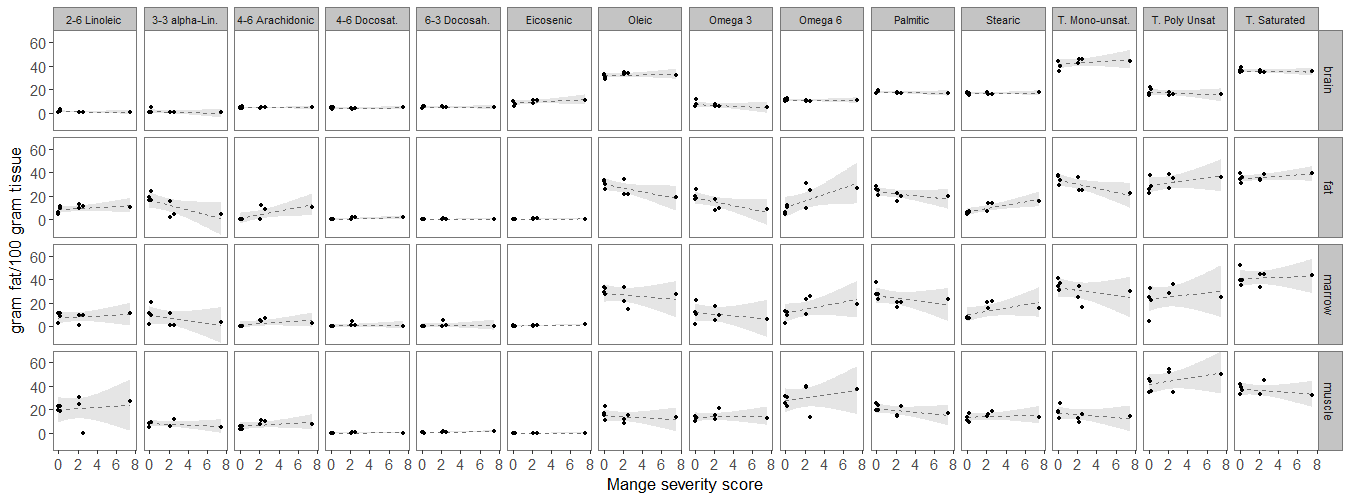
H. Visual representation of the 14 fatty acids with the best explanatory power in relationship to mange score.
